# High-Efficient Flame-Retardant Finishing of Cotton Fabrics Based on Phytic Acid

**DOI:** 10.3390/ijms24021093

**Published:** 2023-01-06

**Authors:** Wan-Meng Song, Li-Yao Zhang, Ping Li, Yun Liu

**Affiliations:** College of Textiles and Clothing, Institute of Functional Textiles and Advanced Materials, National Engineering Research Center for Advanced Fire-Safety Materials D & A (Shandong), State Key Laboratory of Bio-Fibers and Eco-Textiles, Qingdao University, Qingdao 266071, China

**Keywords:** cotton fabrics, phytic acid, flame retardancy, anti-wrinkle properties

## Abstract

In this study, an efficient phosphorus-containing flame retardant, PAPBTCA, was synthesized from phytic acid, pentaerythritol, and 1,2,3,4-butane tetracarboxylic acid, and its structure was characterized. PAPBTCA was finished on cotton fabrics by the pad-dry-curing process, and the flame retardancy, flame-retardant durability, and wrinkle resistance of the obtained flame-retardant fabrics were investigated. It should be noted that the heat release rate value of the flame-retardant cotton fabrics treated with 200 g/L PAPBTCA decreased by 90% and its excellent flame retardancy was maintained after 5 washing cycles. Meanwhile, the wrinkle resistance of flame-retardant cotton fabrics has been significantly improved. In addition, compared with the control, the breaking force loss of PAPBTCA-200 in the warp and weft directions was 24% and 21%, respectively. This study provides a new way to utilize natural phosphorus-based flame retardants to establish multifunctional finishing for cotton fabrics.

## 1. Introduction

Cellulose, as one of the natural polymer materials, is widely used because it is green, renewable, and abundant in resources. Cotton fabrics are favored among natural cellulose fabrics because of their comfort, breathability, and softness [1,2,3]. Due to their composition unit of glucose, cotton fabrics have a low limiting oxygen index (LOI) value of only 18%, making them flammable, which limits the application of cotton fabrics in various fields [4]. It has been reported that the annual casualties and economic losses caused by fires are unimaginable, so people’s awareness of fire safety is increasing, and the development of fire safety textile research is also increasing [5,6]. However, considering the ongoing advancements in science and technology and the increasing needs of people, only the flame-retardant function of cotton fabrics cannot meet the expectations of people in many aspects [7]. Therefore, making cotton fabrics versatile on the basis of excellent flame retardancy is a hot spot for many researchers [8,9,10].

Currently, phosphorus-based flame retardants with good flame retardancy are a common choice for flame-retardant natural cellulose fabrics [11,12]. When cellulose is burned at higher temperatures, many phosphorus-based flame retardants dehydrate and decompose to produce metaphosphoric acid or polyphosphoric acid. Metaphosphoric acid or polyphosphoric acid promotes cellulose to dehydrate and carbonize into char residue [13,14]. However, partial phosphorus-based flame retardants also have problems, including a waste of resources and environmental pollution. As the world’s major environmental problems are becoming serious, environmentally friendly flame retardants free of formaldehyde and halogens are becoming increasingly popular [15,16,17]. Because of its excellent biocompatibility, environmental friendliness, renewability, and degradability, phytic acid (PA) containing 28% phosphorus in its structure, an organic phosphonic acid produced from plants, has become a popular flame retardant in recent years [18]. Ma et al. used PA and urea to finish cotton fabrics to provide them with flame retardancy, and the finished fabrics had excellent flame retardancy and durability [19]. Liu et al. also prepared flame retardants by reacting PA with urea and finishing them on lyocell fabrics. The LOI value of finished lyocell fabrics reached 39.2% and was maintained at 29.7% after 30 washing cycles. This study achieved a combination of highly efficient flame retardancy and good durability [20]. However, PA is highly acidic and can cause great damage to the mechanical properties of cellulose fabrics when used alone as a flame retardant [21]. To solve these problems, it is a feasible method to introduce a suitable carbon-forming agent into the PA system to reduce the damage that PA brings to cellulose fabrics. Pentaerythritol (PER) is a small molecule carbon-forming agent that was used in the early days, but its water solubility and easy precipitation made it ineffective when used in an intumescent flame-retardant system (IFR). Zhu’s team designed an organic–inorganic hybrid phosphorus–boron nitride synergistic flame retardant using PA, PER, and boric acid. This system provided excellent flame retardancy and durability to cotton fabrics with lower weight gain [22]. BTCA (1,2,3,4-butane tetracarboxylic acid) has been extensively studied as a polycarboxylic, acid-based, anti-wrinkle finishing agent [23]. Since it has four carboxyl groups, it can be used to couple fabrics and flame retardants, and the residual BTCA during the finishing process can provide an anti-wrinkle finishing effect to cotton fabrics.

Cheng et al. successfully synthesized a flame retardant through PA, PER, and BTCA for wool fabrics, with the flame retardant linked to wool through electrostatic interaction and ester bonding, resulting in good flame-retardant properties for wool fabrics after 20 washing cycles [24]. This work was excellent but the system was not applied to cotton fabrics. In order to investigate whether this system also has a bright effect on the durability of flame-retardant cotton fabrics, PA, PER, and BTCA were used to synthesize PAPBTCA. The hydroxyl group in PER is highly reactive, so it can be esterified with the phosphate group in PA to obtain the flame retardant PAP. PAP can be cross-linked with BTCA to form the cross-linkable flame retardant PAPBTCA, and it can react with cotton fabric’s hydroxyl groups to create long-lasting flame-retardant cotton fabrics. A series of characterizations of the synthesized PAPBTCA have been performed and the properties and mechanism of the flame-retardant cotton fabrics have been examined.

## 2. Results and Discussion

### 2.1. FTIR of the Control, PAP-200, and PAPBTCA-200

**Figure 1:**
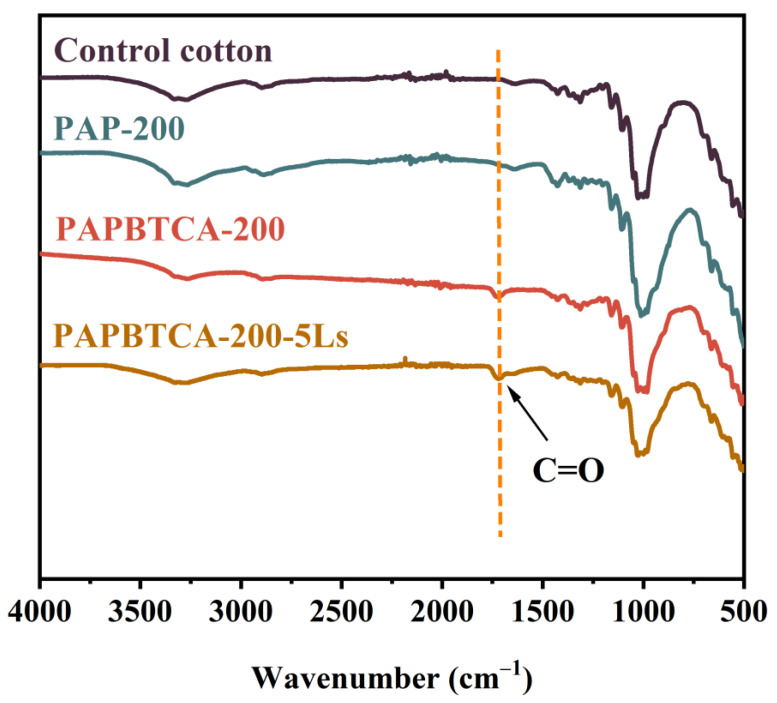
FTIR spectra of the treated and untreated cotton fabrics.

The structure of the control, PAP-200, PAPBTCA-200, and PAPBTCA-200 after 5 washing cycles (PAPBTCA-200-5Ls) was analyzed by FTIR. The results in Figure 1 show that a new characteristic C=O peak at 1724 cm^−1^ appeared in PAPBTCA-200, which was attributed to the ester bond in PAPBTCA and the ester bond formed by the esterification reaction between PAPBTCA and the fabrics [25]. It is noteworthy that PAPBTCA-200-5Ls can still observe a significant C=O peak, and this indicates that there was still PAPBTCA deposited on the surface of the fabrics after 5 washing cycles. The results of FTIR show that PAPBTCA successfully deposited on the surface of cotton fabrics.

### 2.2. Surface Morphology of the Control, PAP-200, and PAPBTCA-200

**Figure 2:**
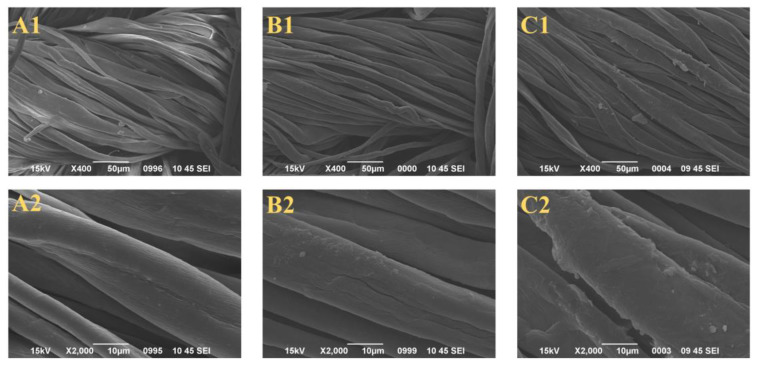
SEM micrographs (×400 and ×2000) of untreated and treated cotton fabrics: Control (**A1**,**A2**), PAP-200 (**B1**,**B2**), PAPBTCA-200 (**C1**,**C2**).

SEM was performed on the control, PAP-200, and PAPBTCA-200 to determine their surface morphology. The resulting pictures are displayed in Figure 2. It is clear that the control has a natural twist, and also an irregular spiral twist in the longitudinal plane. Compared with the control, the surface morphology of PAP-200 and PAPBTCA-200 did not change much, but there was a significant deposition on the surface of the fibers. Compared with PAP-200, PAPBTCA-200 was deposited more obviously on the fabrics, and the cotton fibers became thicker. SEM micrographs proved that the exterior of the fabrics had been successfully coated with flame retardants.

### 2.3. Flame Retardancy

**Figure 3:**
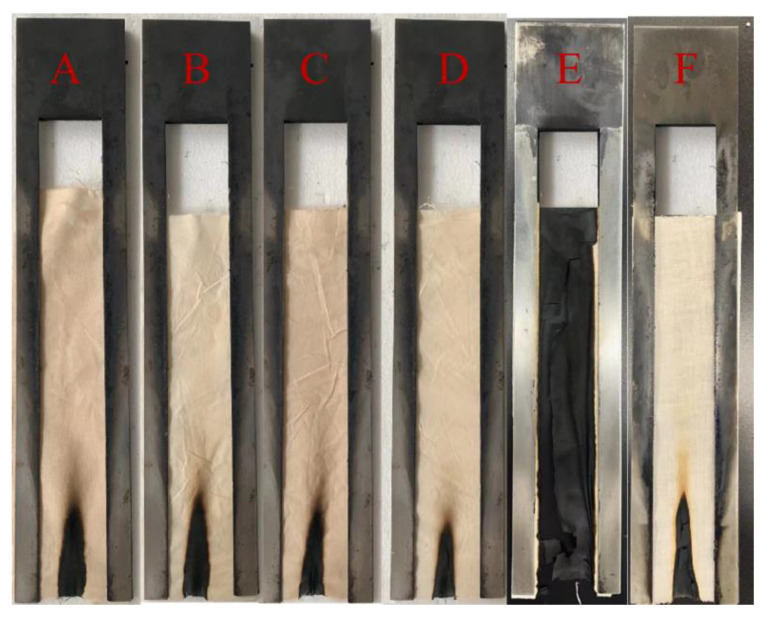
VFT photos of the samples: PAP-100 (**A**), PAPBTCA-100 (**B**), PAP-200 (**C**), PAPBTCA-200 (**D**), PAP-200-5Ls (**E**), PAPBTCA-200-5Ls (**F**).

To investigate the flame retardancy of cotton fabrics treated with varying concentrations of PAP and PAPBTCA, respectively, VFT and LOI tests were used. The images and data obtained from the test are presented in Figure 3 and Table 1. The results show that the addition of BTCA had no discernible impact on the flame-retardant effect of PAP. Moreover, PAP-200 lost its flame-retardant property after the washing durability test. PAPBTCA-100 had a good flame-retardant effect with 17.3% weight gain and the LOI value reached 29.5%, while the LOI value of PAPBTCA-200 reached 31.5% when the weight gain was 20.3%. After 5 washing cycles, the weight gain was still 16.8% and the LOI value was 29.3%, which also passed the VFT test. This indicates that PAPBTCA-200 improves the washing durability of flame-retardant finishing due to the cross-linking effect of BTCA.

### 2.4. Thermal Stabilities

**Figure 4:**
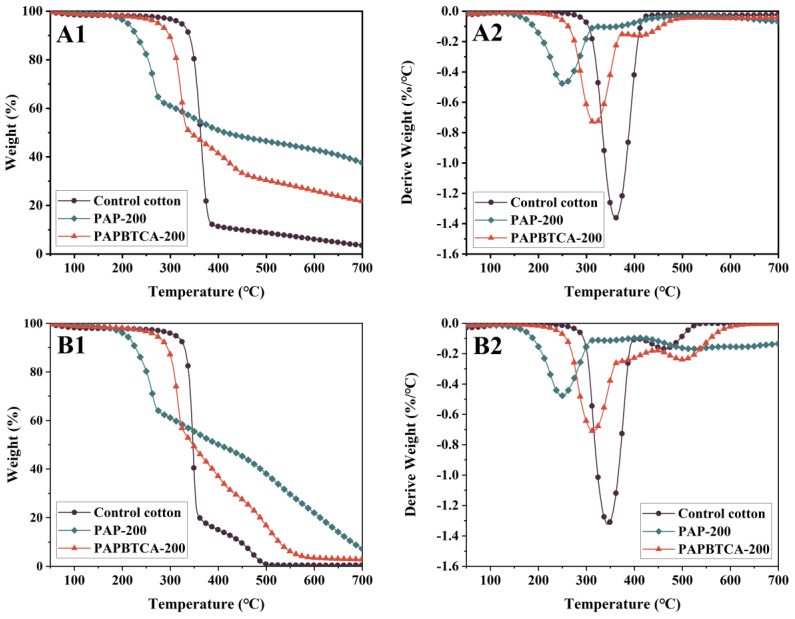
TG and DTG curves of the control, PAP-200, and PAPBTCA-200 in N_2_ (**A1**,**A2**) and air (**B1**,**B2**).

TG analysis was performed to examine the effect of PAP and PAPBTCA on the thermal degradation process of cotton fabrics. Figure 4 displays the samples’ TG and DTG curves. The data derived from these curves are summarized in Table 2.

The samples were significantly degraded between 250 and 450 °C with a maximum loss of weight, and this was brought on by the dehydration and carbonization of cotton fibers, which resulted in the production of volatiles and aliphatic char residues [26,27]. As the temperature increased, the cotton fabrics experienced further thermal degradation, releasing CO_2_ and CO, etc. In a N_2_ atmosphere, R_max2_ was observed at 345 °C and 409 °C for PAP-200 and PAPBTCA-200, respectively, which may be due to the thermal degradation of unstable intermediates. The char residues of PAP-200 and PAPBTCA-200 increased to 37.68% and 21.28%, respectively, at 700 °C compared with 3.65% char residue for the control. The altered thermal degradation pathway of cotton fabrics facilitated the formation of protective and stable char residue. Phosphoric acid or polyphosphate produced from the thermal degradation of PA aided in the dehydration and carbonization of cotton fabrics to produce stable char residue. The results show that the addition of flame retardant effectively inhibited the thermal decomposition process of cotton fabrics and improved the char formation ability of cotton fabrics in the high-temperature range [28]. The initial decomposition temperatures (T_5%_) of PAP-200 and PAPBTCA-200 were also lower than the control during the thermal oxidative degradation in air atmosphere. Compared with the control, the earlier R_max1_ of PAP-200 and PAPBTCA-200 was caused by the earlier decomposition of PAP and PAPBTCA, while the later R_max2_ reflected that the formed intermediates were not thermal stable to produce the more thermal stable char residue [28]. However, they had fewer char residues at 700 °C, with values of only 7.24% and 2.86%, and those were related to the subsequent thermal oxidation of the generated char residues in air.

In summary, the addition of PAPBTCA reduced the thermal stability of cotton fabrics at low temperatures, but increased the number of char residues at high temperatures. These results indicated that PAP and PAPBTCA enhanced the production of stable char residues during the thermal degradation of cotton fabrics, and that increased their thermal stability in the high-temperature range. From Figure 4 and Table 2, the results show that the maximum thermal decomposition rate (R_max_) values of both PAP-200 and PAPBTCA-200 were reduced, and that the amount of char residue was much higher than that of the control; however, the advantage of PAPBTCA-200 was smaller and the amount of char residue was less, compared with those of PAP-200. This may be due to the lower P content of PAPBTCA compared with that of PAP in the case of similar weight gain, resulting in a relatively weaker flame-retardant efficiency.

### 2.5. Burning Behaviors

**Figure 5:**
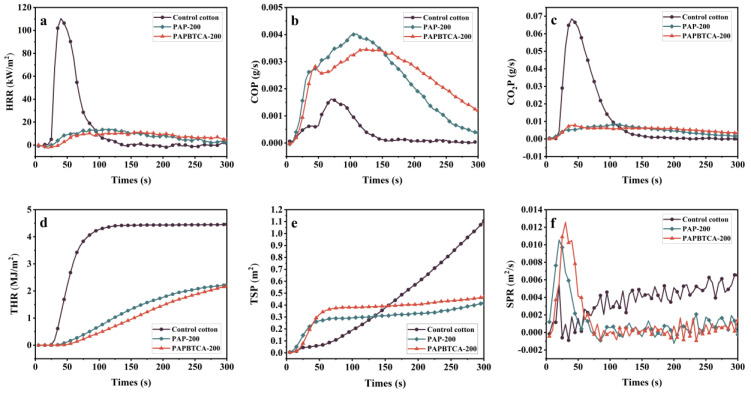
HRR (**a**), COP (**b**), CO_2_P (**c**), THR (**d**), TSP (**e**), and SPR (**f**) curves of the control, PAP-200, and PAPBTCA-200.

CCT can simulate the real situation of fires and examine the burning characteristics of the control, PAP-200, and PAPBTCA-200. The samples’ curves from CCT are displayed in Figure 5, while Table 3 displays the CCT data.

The time to ignition (TTI) of the control was approximately 18 s, while PAP-200 and PAPBTCA-200 were not ignited to produce a flame at the tested heat flux. This situation showed that the treated cotton fabrics were hard to ignite. The peak heat release rate (PHRR) of the control was 110 kW/m^2^, comparatively, and PHRR values of PAP-200 and PAPBTCA-200 were decreased by 88% and 90%, respectively. Furthermore, the average heat release rate (Av-HRR) values of PAP-200 and PAPBTCA-200 were also reduced by 54% and 57%, respectively. This indicates that PAP and PAPBTCA effectively inhibited heat release during the burning process, while the lower PHRR value means that the burning process is more likely to be interrupted. The total heat release (THR) value of the control was 4.5 MJ/m^2^, and those of PAP-200 and PAPBTCA-200 were 2.3 and 2.1 MJ/m^2^, respectively. In comparison, they dropped by 49% and 53%, respectively. In summary, PAP and PAPBTCA reduced the heat-release properties of cotton fabrics.

One of the main factors that contributes to fire deaths is smoke [29,30]. Current studies also focus on the smoke suppression capabilities of flame-retardant materials [31]. As can be observed from Figure 5 and Table 3, the total smoke production (TSP) values of PAP-200 and PAPBTCA-200 decreased from 2.8 m^2^ to 0.4 and 0.5 m^2^, respectively. This indicates that the addition of PAP and PAPBTCA has a good smoke suppression effect, and PA facilitates the carbonization and dehydration of cellulose fibers to create a barrier, resulting in the inhibited effect on the smoke release at the condensed phase [32]. This also indicates that PAP and PAPBTCA play a key role in suppressing smoke released during burning. In addition, the information presented by the CO production rate (COP) and CO_2_ production rate (CO_2_P) in Figure 5 cannot be ignored. It is obvious that the COP values of PAP-200 and PAPBTCA-200 were significantly increased, while the CO_2_P values were seriously decreased. Those phenomena might be caused by the flame retardants promoting the decomposition of cotton fabrics into char residue, and the dense and stable char layers isolating the external heat and air, resulting in the incomplete burning of the fabrics. This was also attributed to the significantly higher char residue of PAP-200 and PAPBTCA-200. The fire growth index (FIGRA) indicates the degree of danger of a material in a fire, which can be found by PHRR/T_PHRR_. A decrease in FIGRA can indicate that people have a greater chance to escape in the event of a fire. From the test results, it can be seen that the FIGRA values of PAP-200 and PAPBTCA-200 are significantly lower compared with the control. The above results show that PAP-200 and PAPBTCA-200 have higher flame retardancy and smoke suppression performance.

**Figure 6:**
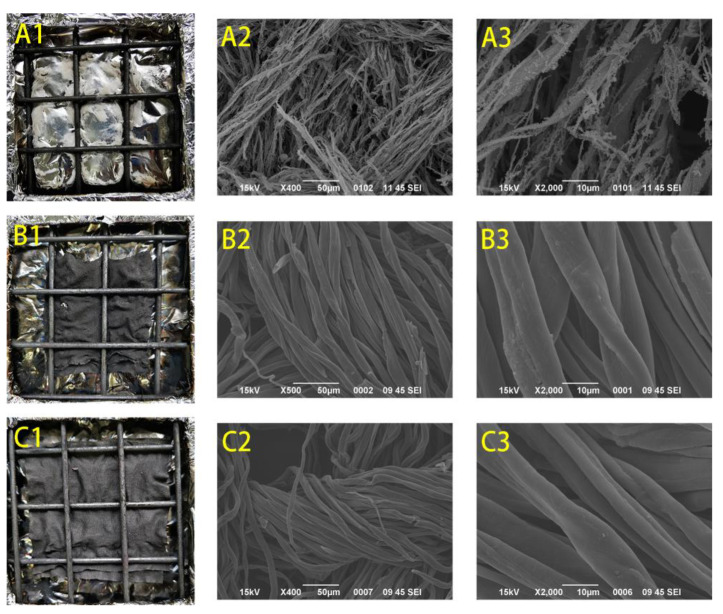
SEM micrographs (×400 and ×2000) of the control (**A1**–**A3**), PAP-200 (**B1**–**B3**), and PAPBTCA-200 (**C1**–**C3**) after CCT.

The char residues after CCT for the control, PAP-200, and PAPBTCA-200 were imaged by SEM, as presented in Figure 6. As observed in Figure 6(A1–A3), after CCT, the control was turned into messy and fragile ash that did not maintain its fabric structure. By comparison, the char residue of PAP-200 and PAPBTCA-200 showed a significant difference. In Figure 6(B1–C3), the char residue of treated cotton fabrics had only some fractures and cracks caused by heat, but they generally maintained their original fiber structure and relatively complete shape. Moreover, the char residual morphology of treated cotton fabrics was relatively similar. The relatively undamaged char residue can prevent further burning by blocking heat and oxygen, which also reduces THR derived from CCT.

### 2.6. Flame-Retardant Mechanism

#### 2.6.1. Analysis of the Structure of Char Residues

**Figure 7:**
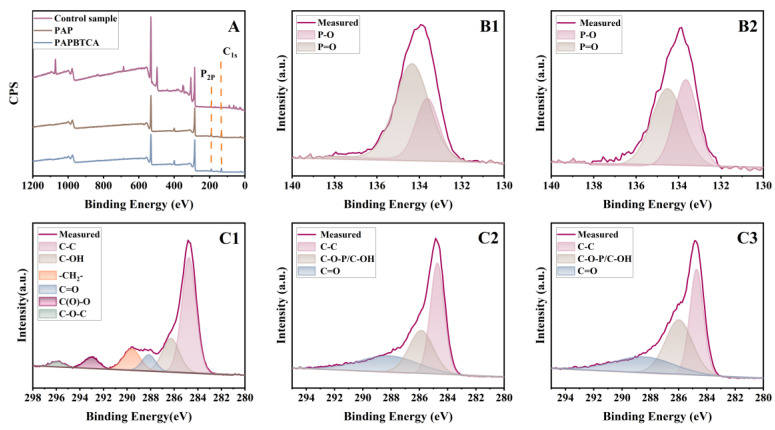
XPS spectra of char residue (**A**), P2p of PAP-200 (**B1**), P2p of PAPBTCA-200 (**B2**), C1s of control cotton (**C1**), C1s of PAP-200 (**C2**), and C1s of PAPBTCA-200 (**C3**).

The elemental composition and different types of chemical bonds of the char residue after CCT were examined using XPS. C and P elements in char residues of the control, PAP-200, and PAPBTCA-200 are shown in Figure 7. In Figure 7A, compared with the control, PAP-200 and PAPBTCA-200 showed a new P2p peak at 130.0 eV. This indicates that the char residue contained P element. They were the characteristic peaks of P-O-C groups in the cross-linked char residue produced by the thermal degradation of the flame retardant and the P=O in the phosphate structure [33]. In Figure 7(C1–C3), the peaks at 284.6, 285.8, and 288.3 eV were responsible for C-C, C-O-P/C-OH, and C=O groups, respectively [34,35]. C elements in char residue of treated cotton fabrics mainly contained C-C, C-O-P, and C=O bonds, meaning that the flame retardant participated in the creation of char residue. Based on the previous research, PAP and PAPBTCA formed phosphoric acid or polyphosphate acid during the thermal degradation process. These two substances stimulated the carbonization and dehydration of cellulose, and also the formation of char residues. These char residue can not only isolate oxygen and heat sources but also restrain the further burning of cotton fabrics.

**Figure 8:**
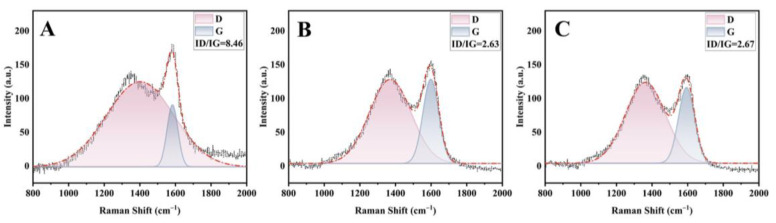
Raman spectra of char residues of control cotton (**A**), PAP-200 (**B**), and PAPBTCA-200 (**C**) after CCT.

The char residue after CCT of PAP-200 and PAPBTCA-200 was also examined by Raman spectra, shown in Figure 8. The signature D and G peaks in Raman spectra were positioned at approximately 1300 cm^−1^ and 1580 cm^−1^, respectively. The I_D_/I_G_ value was calculated by the area ratio of peak D to peak G. The density of graphite imperfections is represented by the I_D_/I_G_ value, which is related to the severity of structural flaws in graphite. The I_D_/I_G_ value of char residue from the control was 8.46, while those of PAP-200 and PAPBTCA-200 were 2.63 and 2.67, respectively. The I_D_/I_G_ values of PAP-200 and PAPBTCA-200 were much lower compared with that of the control, demonstrating that the completed char residues had a higher degree of graphitization. In addition, the char residue with higher degrees of graphitization improved the thermal stability to stop heat transmission, thus enhancing the flame retardancy.

#### 2.6.2. TG-FTIR

**Figure 9:**
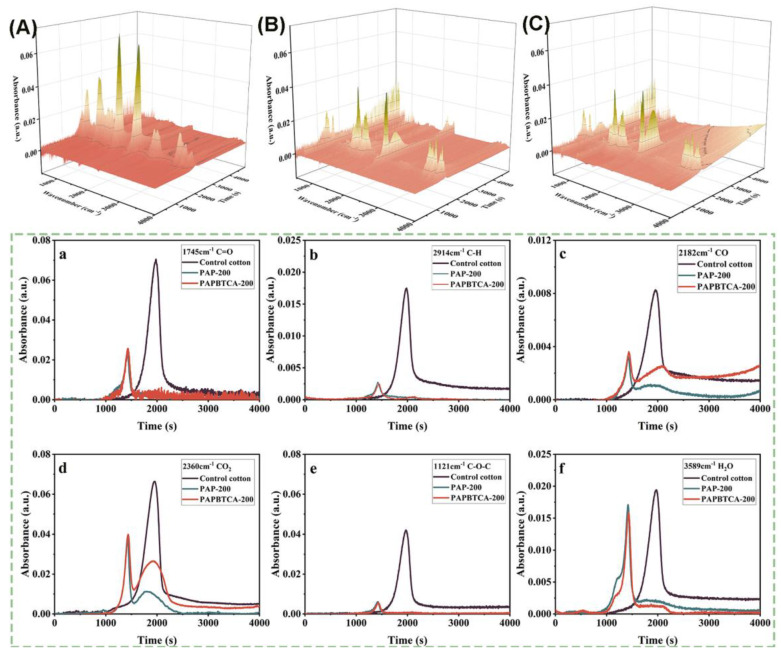
Three-dimensional TG-FTIR spectra of the control cotton (**A**), PAP-200 (**B**), and PAPBTCA-200 (**C**), and the absorption intensities of selected peaks during the thermal degradation process of samples (**a**–**f**).

In order to analyze the flame-retardant mechanism of this system, the FTIR 3D spectra of the control, PAP-200, and PAPBTCA-200 obtained from TG-FTIR are displayed in Figure 9. From Figure 9A–C, the absorption peaks of the control, PAP-200, and PAPBTCA-200 are similar. However, most absorption peaks of PAP-200 and PAPBTCA-200 were weaker than those of the control. The –OH groups were responsible for the 3589 cm^−1^ peaks, which can be found in gaseous water and other substances containing –OH groups. The absorbance of the C-H groups appeared at approximately 2914 cm^−1^ [21], with the absorption intensities of the C-H groups lower than those of the control. Hydrocarbons are very common flammable chemicals, and these results showed that fewer hydrocarbons were produced during the thermal decomposition of PAP-200 and PAPBTCA-200. The peak at 2360 cm^−1^ was attributed to CO_2_ stretching vibration, the peak at 1745 cm^−1^ was due to the stretching vibration of C=O groups, and the peak at 1121 cm^−1^ was ascribed to the stretching vibration of C-O-C groups. Figure 9 clearly demonstrates that the absorption intensities of CO_2_ for PAP-200 and PAPBTCA-200 were considerably lower than that of the control. The above results indicate that more carbon remained in the condensed phase. However, the absorption intensities of the C=O and C-O-C groups for PAP-200 and PAPBTCA-200 were also slightly lower than those of the control, clearly showing that PAP and PAPBTCA promote the dehydration and carbonization of cellulose to create char residue rather than the production of flammable volatile gases and CO_2_ [36].

Six spectra from the aforementioned groups are shown in Figure 9a–f, including H_2_O (3589 cm^−1^), C–H (2914 cm^−1^), CO_2_ (2360 cm^−1^), C–O (1745 cm^−1^), CO (2182 cm^−1^), and C–O–C (1121 cm^−1^). Among them, the absorption intensities of CO_2_ for PAP-200 and PAPBTCA-200 were lower than that of the control, which is compatible with the above conclusion of the FTIR 3D spectra. The lower absorption intensities of PAP-200 and PAPBTCA-200 indicate that flammable gases were reduced to prevent the burning. In addition, compared with the control, the degraded product peaks of PAP-200 and PAPBTCA-200 occurred earlier, indicating that the addition of flame retardants catalyzed and accelerated the dehydration and carbonization of cellulose [37,38]. In summary, PAP and PAPBTCA primarily achieved an outstanding flame-retardant effect by inhibiting the production of flammable gases within the gas phase and encouraging the formation of char residue in the condensed phase.

### 2.7. Anti-Wrinkle Performance

The crease recovery angle test was conducted on the control and treated cotton fabrics to analyze whether the crease resistance was improved or not, with Table 4 displaying the pertinent information. As is evident from the crease recovery angle data, in comparison with the control, there were no noticeable changes in the crease phenomenon of PAP-200. However, PAPBTCA-200 showed an increase in crease recovery angle from 160.8° to 184.7°, an increase of 14.9% compared with that of the control. The slow crease recovery angle increased from 168.7° to 230.9°, an increase of 36.9%, indicating that PAPBTCA resulted in an improvement in the crease resistance of the cotton fabrics [39,40]. The improved anti-wrinkle property may be due to the cross-linking of excess BTCA with the –OH groups of the cotton fabrics, which acted as an anti-wrinkle agent [41]. From the results, it is evident that the anti-wrinkle performance of PAPBTCA-200 was significantly improved compared with those of the control and PAP-200.

### 2.8. Breaking Force

**Figure 10:**
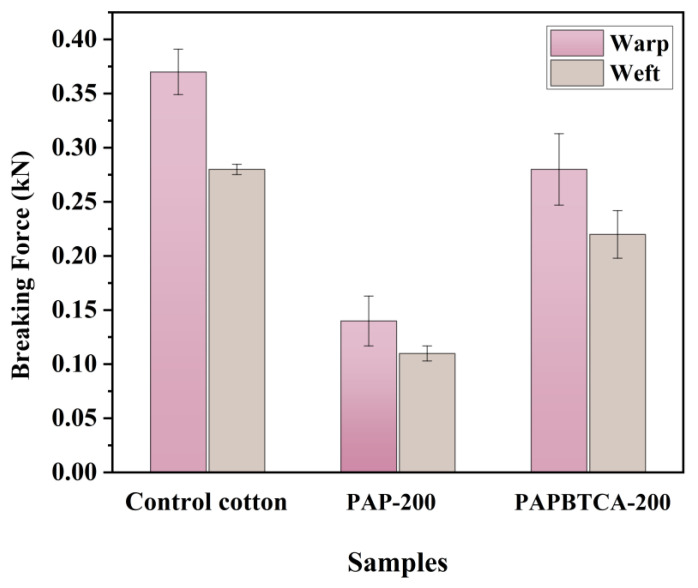
Breaking force of the control, PAP-200, and PAPBTCA-200.

Breaking force was tested for the control, PAP-200, and PAPBTCA-200. The results are shown in Figure 10. Compared with the control, the breaking force of PAP-200 was significantly reduced, with 62% reduction in the warp direction and 61% reduction in the weft direction. However, the breaking force loss of PAPBTCA-200 was alleviated. Compared with the control, the breaking force loss of PAPBTCA-200 in the warp and weft directions was 24% and 21%, respectively. This suggests that the addition of BTCA can further reduce the acidity of PA compared to PAP, thus having a greater certain protective effect on the fabrics’ strength [42]. There was also another reason for this phenomenon: the films formed by PAPBTCA-200 were thicker than the films formed by PAP-200 (shown in Figure 2), making the cotton fibers less prone to breakage.

## 3. Materials and Methods

### 3.1. Materials

Cotton fabrics with 100 g/m^2^ were offered by Qingdao No. 6 Printing and Dyeing Co., Ltd. (Qingdao, China); ammonia and NaOH were supplied by Sinopharm Chemical Reagent Co., Ltd. (Shanghai, China); PA (70% aqueous solution), PER, and BTCA were offered by Macklin Chemical Reagent Co., Ltd. (Shanghai, China). All the chemicals were used as received.

### 3.2. Preparation

#### 3.2.1. Synthesis of PAP and PAPBTCA

PA (0.01 mol, 6.6 g) and PER (0.03 mol, 4.1 g) were mixed in a three-necked flask (250 mL) and heated for 2 h at 130 °C under magnetic stirring to obtain a viscous and little yellow liquid (PAP). The reaction was then performed at 130 °C with magnetic stirring for a further 1 h after the addition of BTCA (0.03 mol, 7.0 g) to the solution. Finally, the transparent liquid became more elastic and yellow. The raw product was freeze-dried and then refined using ethanol. PAPBTCA was prepared according to [24]. Figure S1 shows the relevant characterization of PAPBTCA.

#### 3.2.2. Preparation of Flame-Retardant Cotton Fabrics

**Figure 11:**
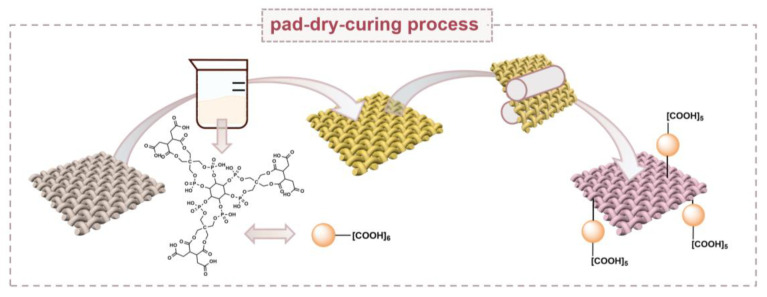
The preparation process of flame-retardant cotton fabrics.

Cotton fabrics with suitable sizes were submerged into 1% NaOH solution at 100 °C for 1 h, washed, and dried at 80 °C. The unpurified PAPBTCA was dissolved in distilled water to obtain 100 g/L and 200 g/L solutions for treating cotton fabrics, which were named PAPBTCA-100 and PAPBTCA-200, respectively. The cotton fabrics were also treated with 100 g/L and 200 g/L of PAP solutions as a comparison and referred to as PAP-100 and PAP-200, respectively. Then, 3 wt% sodium hypophosphite was added to the flame-retardant solutions as a catalyst. The cotton fabrics were soaked in the finishing solution for 30 min, with two paddings, then dried at 80 °C for 3 min and, lastly, the treated cotton fabrics were cured at 170 °C for 3 min. The samples treated with PAPBTCA were thoroughly washed with deionized water to eliminate unbound PAPBTCA. In the last step, the samples were dried at 80 °C to determine the weight gain. Figure 11 shows the preparation process. Figure S2 shows the reactions that occur in the preparation of flame retardant cotton fabrics.

### 3.3. Characterizations

#### 3.3.1. FTIR

Attenuated total reflection Fourier transform infrared spectroscopy (ATR-FTIR) was collected on a Nicolet IS50 FTIR spectrometer (Thermo Fisher Scientific, Waltham, MA, USA) to measure the chemical structure of PA, PAP, PAPBTCA, the control, PAP-200, and PAPBTCA-200 in a spectrum range of 4000∼500 cm^−1^ and a resolution of 2 cm^−1^. The detailed results of characterized PAPBTCA are presented in supporting information.

#### 3.3.2. NMR

A Bruker AVANCE III HD 400 MHz spectrometer (Bruker, Bremen, Germany) was used to measure the ^31^P nuclear magnetic resonance (^31^P NMR) with DMSO as the solvent. The detailed results of characterized PAPBTCA are presented in supporting information.

#### 3.3.3. ICP-OES

An inductively coupled plasma emission spectrometer (ICP-OES) (PE Avio 200, USA) was used to detect the P element content of PAPBTCA. Quantitative analysis of elements was obtained by accepting emission spectra at different wavelengths.

#### 3.3.4. SEM

A scanning electron microscope (SEM) analyzer (Tescan Vega 3, TESCAN, Brno, Czech Republic) with an accelerating voltage of 10 kV was used to image the surface morphology of the control, PAP-200, and PAPBTCA-200, before and after the cone calorimeter test (CCT). Due to the SEM test, the sample surface received a gold sputtering treatment.

#### 3.3.5. Vertical Flame Test

The vertical flame test (VFT) was conducted using an LFY-601A vertical flame tester (Shandong Textile Science Research Institute, Qingdao, China). Based on GB/T 5455-2014, the samples were cut into 300 × 80 mm^2^ segments and ignited for 12 s by a 40 ± 2 mm flame. Each sample was examined at least five times.

#### 3.3.6. LOI

A JF-5 oxygen index gauge (Beijing Avic Times Instrument Equipment Co., Ltd., Beijing, China) was used to conduct the limiting oxygen index (LOI) test. According to GB/T 5454-1997, the samples were processed into 150 × 58 mm^2^ segments.

#### 3.3.7. Washing Durability Test

The washing procedure of all samples was performed according to AATCC Test Method 61-2003 test NO.1A. The treated fabrics were washed using the oscillating bath method. The fabrics to be washed were impregnated in conical bottles containing 0.37 wt% detergent. Next, the conical flasks were transferred to an oscillating water bath (40 °C) and every 45 min of oscillation was determined as 5 washing cycles.

#### 3.3.8. TG

The thermal stability of the samples was tested using a STA6000 TG (Perkin-Elmer Ltd., Waltham, MA, USA). Under nitrogen and air atmosphere, respectively, the temperature range was 50–700 °C, the heating rate was 10 °C/min, and the flow rate was 25 mL/min.

#### 3.3.9. CCT

A cone calorimeter (Fire Testing Technology, East Grinstead, UK) with a heat flux of 35 kW/m^2^ was used to examine the burning behaviors of the samples. The samples were cut into 10 × 10 mm^2^ segments according to the ISO 5660-1. At least three evaluations of each sample were performed.

#### 3.3.10. XPS

X-ray photoelectron spectroscopy (XPS) was conducted through an XSAM 800 spectrometer (Kratos Co., Stretford, UK), with 1486.6 eV Al Kα excitation radiation, 12 kV operating voltage, and 15 mA working current selected for this test. The C 1s line was located at the binding energy of 284.0 eV.

#### 3.3.11. Raman Spectra

A Laser Confocal Raman Spectrometer (Thermo Scientific DXR2, Waltham, MA, USA) was used to obtain the Raman spectra of the remaining char residue after CCT.

#### 3.3.12. TG-FTIR

A Perkin-Elmer STA 6000 thermogravimetric analyzer (TG) (Perkin-Elmer Ltd., Waltham, MA, USA) fitted with a Perkin-Elmer Fourier transform infrared spectrometer (FTIR) (Perkin-Elmer Ltd., Waltham, MA, USA) was used. The temperature range for the TG-FTIR test was 40 to 750 °C, with a heating rate of 10 °C/min and a flow rate of 50 mL/min, respectively, in nitrogen atmosphere. FTIR was calibrated to have a spectrum range of 4000 to 500 cm^−1^ and a resolution of 2 cm^−1^.

#### 3.3.13. Anti-Wrinkle Performance Test

The crease recovery property of samples was tested by an LFY-210A/B Fabric crease recovery tester (Qingdao Shanfang Instrument Co., Ltd., Qingdao, China).

#### 3.3.14. Breaking Force

The breaking force was tested on an Instron 5967 electromechanical universal testing machine (Instron Limited, Co., Norwood, MA, USA). The samples were prepared into 300 × 60 mm^2^ segments based on GB/T 3923.1-2013. Each sample was evaluated at least five times.

## 4. Conclusions

In this study, an environmentally friendly, reactive, and highly efficient flame retardant, PAPBTCA, was successfully synthesized by PA, PER, and BTCA. The flame retardant was grafted onto the surface of the cotton fabrics by reacting with the hydroxyl groups of the cotton fabrics and had certain washing durability. Compared with that of the control, the HRR value of PAPBTCA-200 was reduced by 90%, while the smoke release was also significantly reduced. XPS and TG-FTIR tests showed that PAPBTCA had a condensed-phase flame-retardant mechanism. In addition, the crease recovery angle of cotton fabrics after PAPBTCA treatment was significantly improved. Compared with PAP-200, the PAPBTCA-200 breaking force retention has significantly improved. This study shows that biomass containing phosphorus flame retardants has great potential for multifunctional finishing applications.

## Figures and Tables

**Table 1:** Flame-retardant performance of the samples.

Samples	Concentration of Flame Retardants (g/L)	Weight Gain (%)	Afterflame Time (s)	Afterglow Time (s)	Damaged Length (mm)	LOI (%)
Control	/	0.0	17	34	300	18.0
PAP-100	100	15.9 ± 1.1	0	0	77 ± 5	29.7
PAPBTCA-100	100	17.3 ± 1.5	0	0	79 ± 6	29.5
PAP-200	200	21.2 ± 0.4	0	0	71 ± 4	32.6
PAPBTCA-200	200	20.3 ± 1.4	0	0	62 ± 5	31.5
PAP-200-5Ls	200	3.2 ± 0.3	6	0	300 ± 0	20.5
PAPBTCA-200-5Ls	200	16.8 ± 0.7	0	0	98 ± 6	29.3

**Table 2:** Thermogravimetric analysis data of the control, PAP-200, and PAPBTCA-200 in N_2_ and air.

Atmosphere	Samples	T_5%_ (°C)	T_10%_ (°C)	T_max1_ (°C)	R_max1_ (%/°C)	T_max2_ (°C)	R_max2_ (%/°C)	Residue at 700 °C (%)
N_2_	Control cotton	322	367	359	1.36	-	-	3.65
	PAP-200	213	233	249	0.48	345	0.10	37.68
	PAPBTCA-200	288	315	317	0.74	409	0.16	21.82
Air	Control cotton	311	331	343	1.32	462	0.17	0.48
	PAP-200	210	232	248	0.48	521	0.17	7.24
	PAPBTCA-200	283	310	312	0.71	500	0.24	2.86

**Table 3:** CCT data of the control, PAP-200, and PAPBTCA-200.

Samples	TTI (s)	PHRR (kW/m^2^)	Av-HRR (kW/m^2^)	THR (MJ/m^2^)	TSP (m^2^)	FIGRA kW/(m^2^·s)	Residue (wt%)
Control cotton	18 ± 3	110	15.9	4.5	2.8	2.76	5.5
PAP-200	- *	14	7.3	2.3	0.4	0.12	13.1
PAPBTCA-200	- *	11	6.8	2.1	0.5	0.07	20.7

Note: * PAP-200 and PAPBTCA-200 were not ignited.

**Table 4:** Crease recovery angle test of the control, PAP-200, and PAPBTCA-200.

Samples	Crease Sharp Return Angle (°)		Total (°)	Crease Slow Return Angle (°)		Total (°)
	Warp	Weft		Warp	Weft	
Control cotton	79.9	80.9	160.8	83.5	85.2	168.7
PAP-200	81.2	86.1	167.3	86.6	91.1	177.7
PAPBTCA-200	91.6	93.1	184.7	114.7	116.2	230.9

## Data Availability

The data are available upon request.

## Supplementary Materials

The supporting information can be downloaded at: https://www.mdpi.com/article/10.3390/ijms24021093/s1. References [43,44,45] were cited in Supplementary Materials.
