# Supplementary material for: High-Efficient Flame-Retardant Finishing of Cotton Fabrics Based on Phytic Acid
Source: Int J Mol Sci. 2023 Jan 6;24(2):1093. doi: 10.3390/ijms24021093 (PMC9865254; doi:10.3390/ijms24021093)
Supplement: Supplementary file 1 [file ijms-24-01093-s001.zip › ijms-2140839-SI.pdf]

# SUPPORTING INFORMATION

for

# High-Efficient Flame-Retardant Finishing of Cotton Fabrics Based on Phytic Acid

Wan-Meng Song, Li-Yao Zhang, Ping Li and Yun Liu *

**Structure characterizations of PAPBTCA**

The chemical reaction equation of PAPBTCA, the functional groups of PA, PAP, and PAPBTCA examined by FTIR, and the $^{31}$P NMR spectroscopy of PAPBTCA are displayed in Figure S1. Because of the inadequacy of the esterification reaction and the uncertainty of the reacting functional groups, the reaction process cannot be accurately described, and the reaction equation in the ideal state is shown in Figure S1 (A). The FTIR spectrum of PA demonstrated that the bands at 1643 and 991 cm$^{-1}$ were caused by stretching vibrations of P=O and O-P-C [1]. The band at 1163 cm$^{-1}$ was attributed to the presence of C-O groups in PA, PER, and PAPBTCA [2]. The FTIR spectra of PA and PAP were similar because of their similar functional groups. Some additional absorption bands appeared in PAPBTCA compared with those of PA and PAP. It is noteworthy that the strong absorption band at 1722 cm$^{-1}$ was owing to the stretching vibrations of the ester carbonyl bonds formed by the chemical reaction between PER and BTCA [3].

Figure S1 (C) demonstrated the ICP test of the P content of PA and PAPBTCA, and the results of the test showed that the P content of PAPBTCA decreased to 8.9%

compared to the 28% elemental P content of PA. The possible chemical structure of PAPBTCA was further analyzed by $^{31}P$ NMR spectroscopy. The result is displayed in Figure S1 (D). The $^{31}P$ peak of PABTCA appeared around -1.22 ppm, and there were some small peaks around -1.48 ppm, indicating that the synthetic products were not pure. And this was due to the incomplete and inadequate reaction of the esterification reaction [4]. From the FTIR, ICP test and $^{31}P$ NMR spectra results, it can be illustrated that PAPBTCA was successfully synthesized.

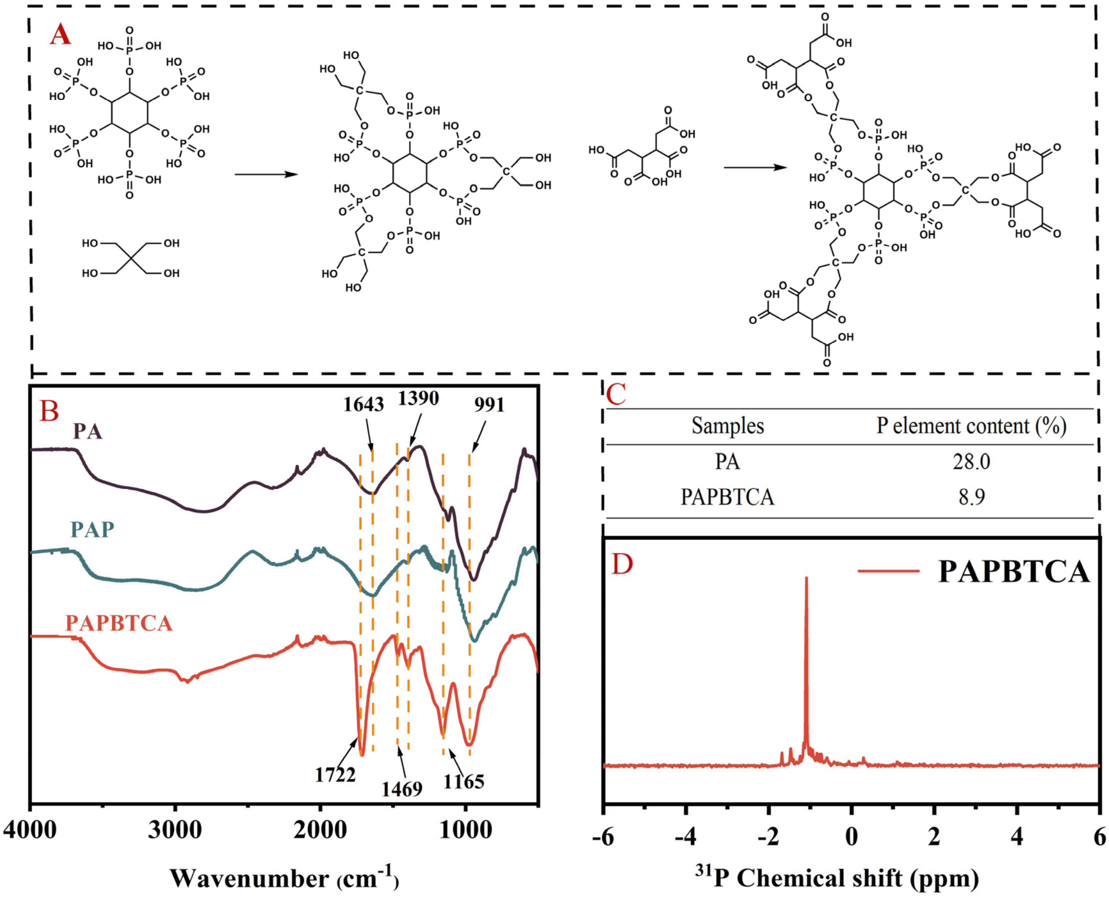


Figure S1. Chemical reaction equation of PAPBTCA (A); FTIR spectra of PA, PAP, and PAPBTCA (B); ICP test of PA and PAPBTCA (C); $^{31}P$ NMR spectra of PAPBTCA (D)

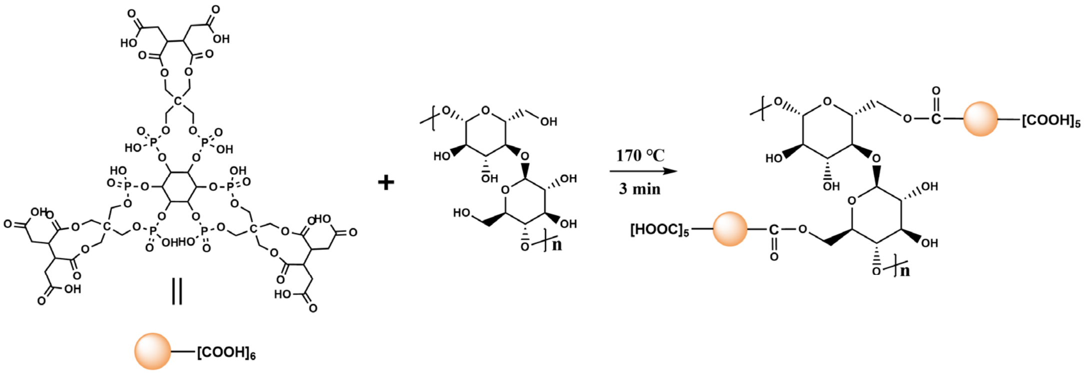

Figure S2. Chemical reaction equation of PAPBTCA-200

## References


[1] G Jiang, J Qiao, H Feng, Application of Phosphoric Acid and Phytic Acid-Doped Bacterial Cellulose as Novel Proton-Conducting Membranes to Pemfc, International Journal of Hydrogen Energy 37(11) (2012) 9182-9192.

[2] X Y Guo, Y Miao, P P Ye, Y Wen, H F Yang, Multi-Walled Carbon Nanotubes in Aqueous Phytic Acid for Enhancing Biosensor, Materials Research Express 1(2) (2014) 025403.

[3] C Q Yang, X L Wang, Formation of Cyclic Anhydride Intermediates and Esterification of Cotton Cellulose by Multifunctional Carboxylic Acids: An Infrared Spectroscopy Study, Textile Research Journal 66(9) (1996) 595-603.

[4] A Bebot-Brigaud, C Dange, N Fauconnier, C Gerard, P-31 Nmr, Potentiometric and Spectrophotometric Studies of Phytic Acid Ionization and Complexation Properties toward $Co^{2+}$, $Ni^{2+}$, $Cu^{2+}$, $Zn^{2+}$ and $Cd^{2+}$, Journal of Inorganic Biochemistry 75(1) (1999) 71-78.
